# A ROS‑responsive liposome for atherosclerosis treatment via combined regulation of lipid metabolism and CD47 blockade

**DOI:** 10.1016/j.ijpx.2026.100626

**Published:** 2026-07-30

**Authors:** Peng Yuan, Shibo Tian, Chao Cui, Chenglu Sun, Chuang Tang, Dengfeng Gao, Lu Lu, Keren Long, Funeng Xu, Gang Shu, Wei Zhang, Xiaoxia Liang, Fanli Kong, Mingzhou Li, Haohuan Li

**Affiliations:** a State Key Laboratory of Swine and Poultry Breeding Industry, College of Veterinary Medicine, Sichuan Agricultural University, Chengdu 611130, China; b Livestock and Poultry Multi-omics Key Laboratory of Ministry of Agriculture and Rural Affairs, College of Animal Science and Technology, Sichuan Agricultural University, Chengdu 611130, China; c College of Veterinary Medicine, Sichuan Agricultural University, Chengdu 611130, China; d State Key Laboratory of Swine and Poultry Breeding Industry, College of Life Science, Sichuan Agricultural University, Chengdu 611130, China

**Keywords:** Foam cells, CD47 dual blockade, Lipid clearance, Atherosclerosis

## Abstract

Atherosclerosis is one of the leading causes of death worldwide. Excessive lipid accumulation and defective efferocytosis trigger massive foam cell formation, which serves as a key driver of atherosclerotic plaque progression. In this work, we developed a phospholipid-based nanocarrier (Ato@LHP) to co-load atorvastatin and a CD47-blocking peptide. This system achieves targeted treatment of atherosclerosis through lipid removal and dual CD47 blockade. We used RAW264.7 cells to build foam cell models *in vitro*, characterized the physicochemical properties of the nanocarrier, and verified its plaque-targeting ability. Six-week-old male ApoE^−^/^−^ mice were fed a high-fat diet for 8 weeks to establish aortic atherosclerosis models, and then were randomly divided into four groups: Saline, Ato@L, Ato@LH, and Ato@LHP. *In vivo* results showed that compared with the Saline group, Ato@LHP treatment reduced aortic plaque area percentage from 28.8% to 11.1%, and the plaque vulnerability index simultaneously decreased from 1.14 ± 0.05 to 0.55 ± 0.04. Mechanistic studies indicated that atorvastatin acts through an endogenous pathway: it blocks the nuclear translocation of NF-κB p50 and reduces CD47 gene transcription. Meanwhile, the nanocarrier responds to reactive oxygen species (ROS) at lesion sites and releases Pep-20 (a CD47-blocking peptide), which directly blocks the CD47/SIRPα interaction *via* an exogenous pathway. These two pathways work together to restore macrophage efferocytosis and accelerate the clearance of apoptotic foam cells. In addition, Ato@LHP clearly reduces lipid accumulation in plaques, relieves local inflammation, and stabilizes atherosclerotic plaques. This strategy of combining dual CD47 blockade with targeted lipid clearance offers a reliable new approach for targeted atherosclerosis treatment.

## Highlights

• Ato@LHP selectively targets atherosclerotic plaques and foam cells *via* CD44 and CD47.
• Dual blockade of CD47 *via* atorvastatin-mediated endogenous suppression and peptide-mediated exogenous inhibition.
• CD47 blockade restores macrophage efferocytosis and reduces apoptotic cell accumulation in plaques.
• Ato@LHP promotes lipid clearance and exerts anti-inflammatory effects to suppress the progression of atherosclerosis.

## Graphical abstract

**Figure:**
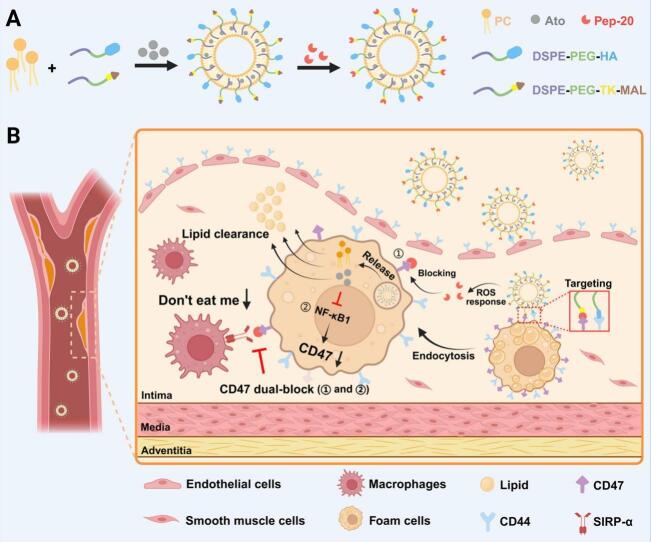

## 1 Introduction

Atherosclerosis is one of the most common and important cardiovascular diseases (CVDs) and is considered a chronic inflammatory disease (Greenhill, 2024; Stojanović et al., 2020). CVDs are the leading cause of death worldwide (Roth et al., 2020). The main characteristics of atherosclerosis include the accumulation of lipids and apoptotic cells (Bäck et al., 2019), a process that forms unstable plaques, further leading to complications such as myocardial infarction, ischaemic cardiomyopathy, stroke and peripheral arterial disease (Libby, 2021).

Macrophages play a key role in lipid accumulation and the clearance of apoptotic cells (Gerlach et al., 2021; Wilson, 2010), and the effective clearance of these cells by macrophages is essential for preventing secondary necrosis and triggering the release of anti-inflammatory cytokines (De Meyer et al., 2024). However, macrophages are gradually converted into foam cells after they engulf many lipoproteins in the intima (Deng et al., 2023; Fernández-Ruiz, 2022; Wang et al., 2019). Lipid accumulation and the formation of foam cells promote apoptosis at plaque sites (Childs et al., 2016), and apoptotic foam cells are not easily removed because of pathological upregulation of the “don't eat me” CD47 molecule (Solanki et al., 2018). The highly expressed CD47 protein specifically binds to signal regulatory protein alpha (SIRPα) on macrophages and inhibits the efferocytosis of apoptotic cells (Kojima et al., 2016; Logtenberg et al., 2020). The continuous accumulation of apoptotic cells and lipids exacerbates the development of atherosclerosis (Adkar and Leeper, 2024). Therefore, reducing foam cell formation and promoting apoptotic cell clearance would be beneficial for treating atherosclerosis and for preventing the development of early atherosclerotic lesions.

Preclinical studies indicate that the use of antibodies and peptides directed against CD47 results in the reactivation of intraplaque efferocytosis and contributes to the prevention of atherosclerosis (Luo et al., 2024; Yu et al., 2020). However, conventional antibodies usually target only the CD47 receptor on the cell surface. Since cells constantly produce new CD47 for surface expression (Berkovits and Mayr, 2015; Whang et al., 2020), the effect may be inadequate. To achieve sufficient CD47 downregulation, a synergistic strategy targeting both endogenous and exogenous CD47 may be effective. Growing evidence indicates that statins not only possess lipid-lowering ability but also downregulate CD47 (Engelen et al., 2022), and statins enhance efferocytosis by inhibiting the nuclear translocation of NF-κB1 p50 and suppressing the expression of the critical ‘don't-eat-me’ molecule, CD47 (Jarr et al., 2022). Atorvastatin can be used to downregulate endogenous CD47 expression *via* the inhibition of its transcription. Therefore, the combination of an antibody or peptide with atorvastatin can better downregulate CD47, which would further promote the restoration of efferocytosis and facilitate the clearance of apoptotic cells.

In this study, we aimed to address the problem of insufficient efferocytosis and excessive lipid deposition, which jointly drive atherosclerotic progression. To achieve this goal, we designed and prepared a multifunctional liposomal nanoplatform (Ato@LHP) for atherosclerosis-targeted therapy. The nanosystem co-delivers atorvastatin and the CD47-blocking peptide Pep-20 (Yu et al., 2021), and employs a ROS-responsive thioketal linker and hyaluronic acid modification to achieve precise plaque targeting and on-demand drug release (He et al., 2024). We hypothesized that the combination of endogenous CD47 transcriptional inhibition by atorvastatin and exogenous CD47/SIRPα blockade by Pep-20 could synergistically restore macrophage efferocytosis, while the combination of atorvastatin and phospholipids could enhance intracellular lipid clearance (LaRosa et al., 2005; Rodrigueza et al., 1998). Unlike single-target strategies, this design combining dual CD47 blockade and lipid clearance provides a new synergistic mechanism to eliminate apoptotic foam cells and inhibit foam cell formation, thereby stabilizing atherosclerotic plaques and blocking disease progression. Overall, this study clarifies the synergistic anti-atherosclerotic effect and underlying mechanism of Ato@LHP, aiming to provide an effective and feasible strategy for targeted treatment of atherosclerosis.

## 2 Experimental

### 2.1 Synthesis of Ato@LHP

Atorvastatin, PC, and DSPE-PEG-TK-MAL were dissolved in trichloromethane, and the solvent was evaporated in a rotary evaporator to obtain a lipid film. The liposome suspension was obtained by hydration with DSPE-PEG-HA solution at 37 °C for 30 min and then by sonication (100 W, 5 min, 3 s operation, 2 s interval) using a probe sonicator. The Pep-20 solution was added to the above liposome mixture, which was stirred overnight at room temperature, at which point homogeneous liposomes were obtained. The obtained product was concentrated with an ultrafiltration membrane (MW = 3000) to remove free drug and peptide, washed three times with PBS, and collected to obtain Ato@LHP.

### 2.2 Synthesis of FITC@LHP and DiD@LHP

These were synthesized in the same manner as Ato@LHP, but atorvastatin was replaced with FITC and DiD.

### 2.3 Characterization of Ato@LHP

The particle size, polydispersity index (PDI) and zeta potential of Ato@L, Ato@LH, and Ato@LHP were analysed with a Zetasizer Nano ZS90 (Malvern, UK). The morphology was observed by TEM (HT7800, Hitachi, Japan). Drug encapsulation efficiency and drug loading were measured *via* a UV–Vis spectrophotometer (UV-2800 A) at 246 nm. The successful conjugation of Pep-20 was verified by high-performance liquid chromatography (HPLC), and its grafting rate was determined. The mobile phase was acetonitrile-water containing 0.1% trifluoroacetic acid; the flow rate was 1 mL min^−1^; and the detection wavelength was 214 nm.

### 2.4 Cellular uptake

RAW 264.7 cells were seeded in 12-well plates (1 × 10^5^ cells per well) and incubated at 37 °C for 24 h. RAW 264.7 cells in the foam cell group were treated with medium containing 40 μg mL^−1^ ox-LDL and 200 ng mL^−1^ LPS for 24 h. HUVECs were seeded in 12-well plates (1 × 10^5^ cells per well) and stimulated with 500 ng mL^−1^ LPS. The culture medium was subsequently replaced with fresh medium containing equal amounts of FITC *via* FITC@L, FITC@LH or FITC@LHP. After 4 h of incubation, the cells were washed with PBS and collected, after which the efficiency of nanoparticle uptake was determined by flow cytometry.

### 2.5 *In Vitro* Blockade of CD47 and macrophage polarization assay

RAW 264.7 cells were seeded in 12-well plates and induced with 40 μg mL^−1^ ox-LDL and 200 ng mL^−1^ LPS, and different groups were treated with PBS, Ato@L, Ato@LH, or Ato@LHP (10 μM atorvastatin) for 24 h. Then, the cells were collected and stained with CD47 and CD11c antibodies and analysed by flow cytometry.

### 2.6 *In vivo* biodistribution analysis assay

Six-week-old male ApoE^−/−^ mice fed a high-fat diet for 8 weeks were injected with equal amounts of DiD@LP or DiD@LHP *via* the tail vein. The mice were sacrificed at 6 h, 12 h, and 24 h after injection. The aorta and major organs (heart, liver, spleen, lung, and kidney) were isolated and washed with PBS. Imaging and quantitative analysis were performed using an IVIS imaging system (Clinx IVScope 8000).

### 2.7 *In vivo* therapeutic assay

Six-week-old male ApoE^−/−^ mice were randomly divided into four groups (Saline, Ato@L, Ato@LH, and Ato@LHP, *n* = 10), and each group was fed a high-fat diet until the end of the treatment period. Treatment began after 8 weeks of high-fat feeding, and the four groups were intravenously administered saline, Ato@L, Ato@LH, or Ato@LHP (5 mg kg^−1^ atorvastatin every three days for 8 weeks). At the end of the treatment period, blood was collected from the mice, and the serum was extracted to determine the concentrations of low-density lipoprotein (LDL), blood glucose (Glu), alanine aminotransferase (ALT), and aspartate aminotransferase (AST). When each mouse was sacrificed, the aorta was isolated for oil red O staining, and the heart arterial roots were collected to obtain paraffin sections, which were used for the following: haematoxylin and eosin (H&E) staining, Masson's trichrome (Masson) staining, immunofluorescence staining and immunohistochemical staining for CD68, alpha smooth muscle actin (α-SMA), matrix metalloproteinase-9 (MMP-9), interleukin-1 beta (IL-1β), tumour necrosis factor-alpha (TNF-α). The heart, liver, spleen, lung and kidney were also collected for H&E staining.

### 2.8 Statistical analyses

Statistical analyses were performed using GraphPad Prism 9 (GraphPad, Inc.). All data are presented as mean ± standard deviation (SD). The Shapiro-Wilk test was used to verify the normality of data distribution, and homogeneity of variances was assessed simultaneously. For data satisfying normal distribution and equal variance, Student's *t*-test, one-way ANOVA followed by Tukey's multiple comparisons test, or two-way ANOVA followed by Šídák's multiple comparisons test were applied. Non-normally distributed datasets were analysed using the Kruskal-Wallis test with Dunn's multiple comparisons test. A *P*-value <0.05 was defined as statistically significant.

The *in vivo* experiment initially included 10 mice per group. All 10 animals were tracked for continuous body weight recording, whereas subgroups of three mice per group were assigned to separate terminal serum biochemistry and histological examinations.

## 3 Results

### 3.1 Preparation and characterization of Ato@LHP

**Fig. 1:**
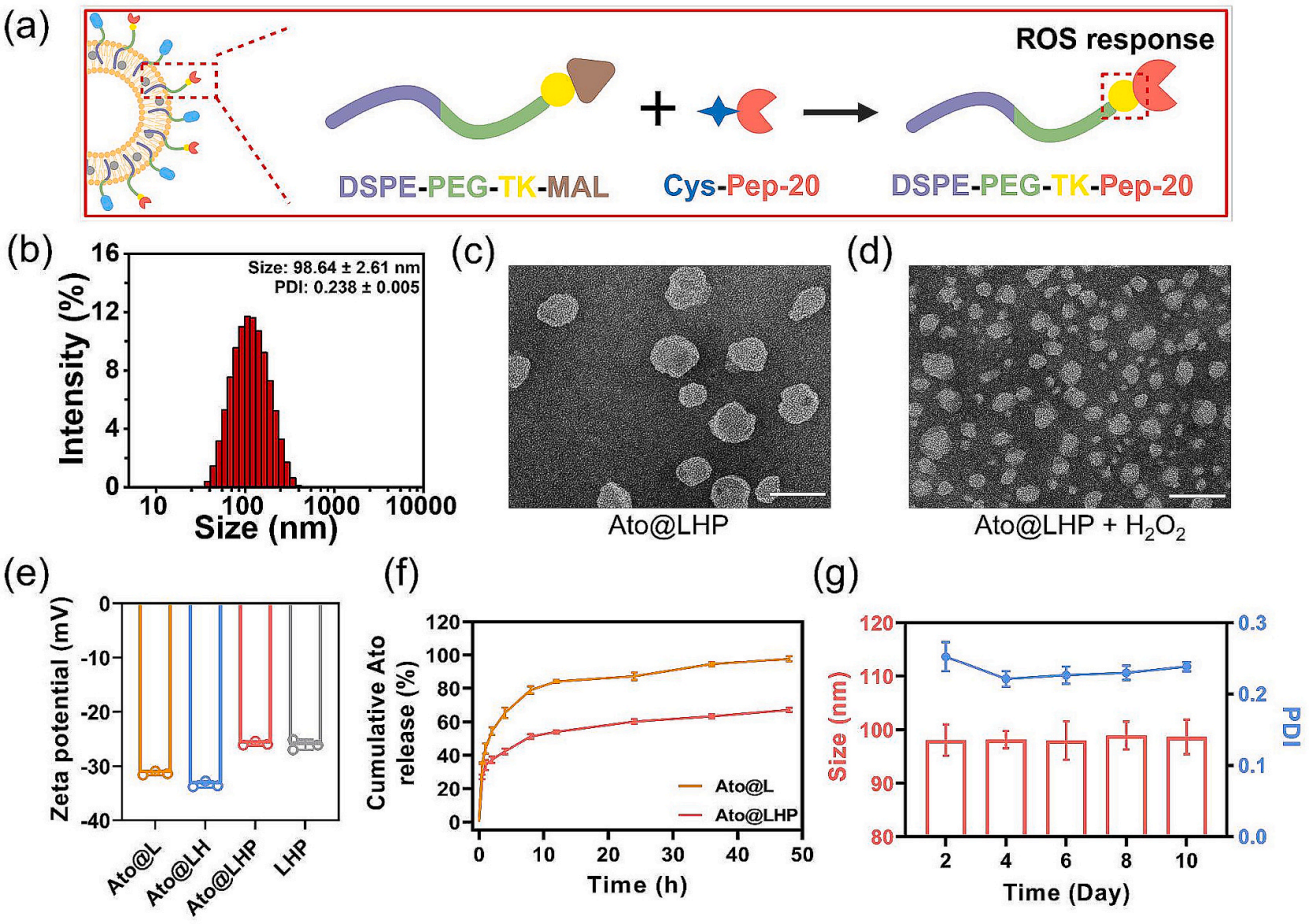
Characterization of Ato@LHP. (a) Schematic representation of Pep-20 surface modification synthesis. (b) Size distributions and PDI of Ato@LHP. (c) TEM image of Ato@LHP. Scale bar = 100 nm. (d) TEM image of Ato@LHP after treatment with 1 mM H_2_O_2_. Scale bar = 100 nm. (e) Zeta potential measurements of Ato@L, Ato@LH, Ato@LHP, and LHP. (f) Cumulative release of Ato from Ato@L and Ato@LHP in PBS. (g) Stability assessment of Ato@LHP particle size and PDI over 10 days in PBS. The data are expressed as the means ± SDs.

In this study, soybean lecithin was used as a carrier to deliver atorvastatin and Pep-20 to the plaque site. The Pep-20 was conjugated to the surface of the liposomes *via* click chemistry (Fig. 1(a)) (Liu et al., 2017). The encapsulation efficiency (EE%) was 82.95%, and the loading efficiency (LE%) was 5.18% of Ato@LHP (Table S1). The particle size distribution of Ato@LHP was determined by dynamic light scattering analysis (Fig. 1(b)). The hydrated particle size was 98.64 ± 2.61 nm with a PDI of 0.238 ± 0.005, and liposomes of this size possess the enhanced permeability and retention (EPR) effect (Fang et al., 2020), which indicates that these liposomes can better penetrate damaged vascular endothelial cells at the lesion site and reach the plaque site for long-term retention. TEM images revealed that Ato@LHP was a uniformly distributed spheroid with a particle size similar to that obtained by DLS (Fig. 1(c)). Zeta potential measurements revealed that Ato@LHP was negatively charged (−25.8 mV) (Fig. 1(e)) and was more stable in the blood circulation (Chen et al., 2020). Based on HPLC characterization, free Pep-20 characteristic peaks were also observed in Ato@LHP (Fig. S1), with a grafting ratio of 7.93% (Fig. S2), which indicates the successful grafting of Pep-20.

To verify the responsiveness of Ato@LHP to ROS, 1 mM hydrogen peroxide was used to simulate the ROS-overexpressing plaque microenvironment. The morphology of Ato@LHP was changed and small fragments were sloughed in the ROS-overexpressing plaque environment (Fig. 1(d)). The HPLC results indicated that the characteristic absorption peak of Pep-20 shifted (Fig. S1), possibly due to exposure of the MAL group after the TK response. Taken together, these results demonstrate that Pep-20 can be released in response to a plaque microenvironment with ROS overexpression.

Furthermore, compared with Ato@L, Ato@LHP exhibited a superior sustained release profile. Based on the cumulative release rate of the drugs, at 48 h, 34.7% of the atorvastatin still remained within Ato@LHP, whereas Ato@L retained only 4.9% (Fig. 1(f)). In addition, Ato@LHP showed good stability in PBS, with no significant change in particle size or PDI after storage at 4 °C for 10 days (Fig. 1(g)).

In addition, Ato@L and Ato@LH were prepared as controls and characterized by particle size, PDI, zeta potential, and TEM (Fig. 1(e), Fig. S3, and Fig. S4). Overall, we prepared a nanodrug with a suitable particle size for the EPR effect that could release Pep-20 in response to ROS at the plaque site, with sustained release and stable placement of the drug.

### 3.2 Targeting properties of atherosclerotic plaques *in vitro* and *in vivo*

**Fig. 2:**
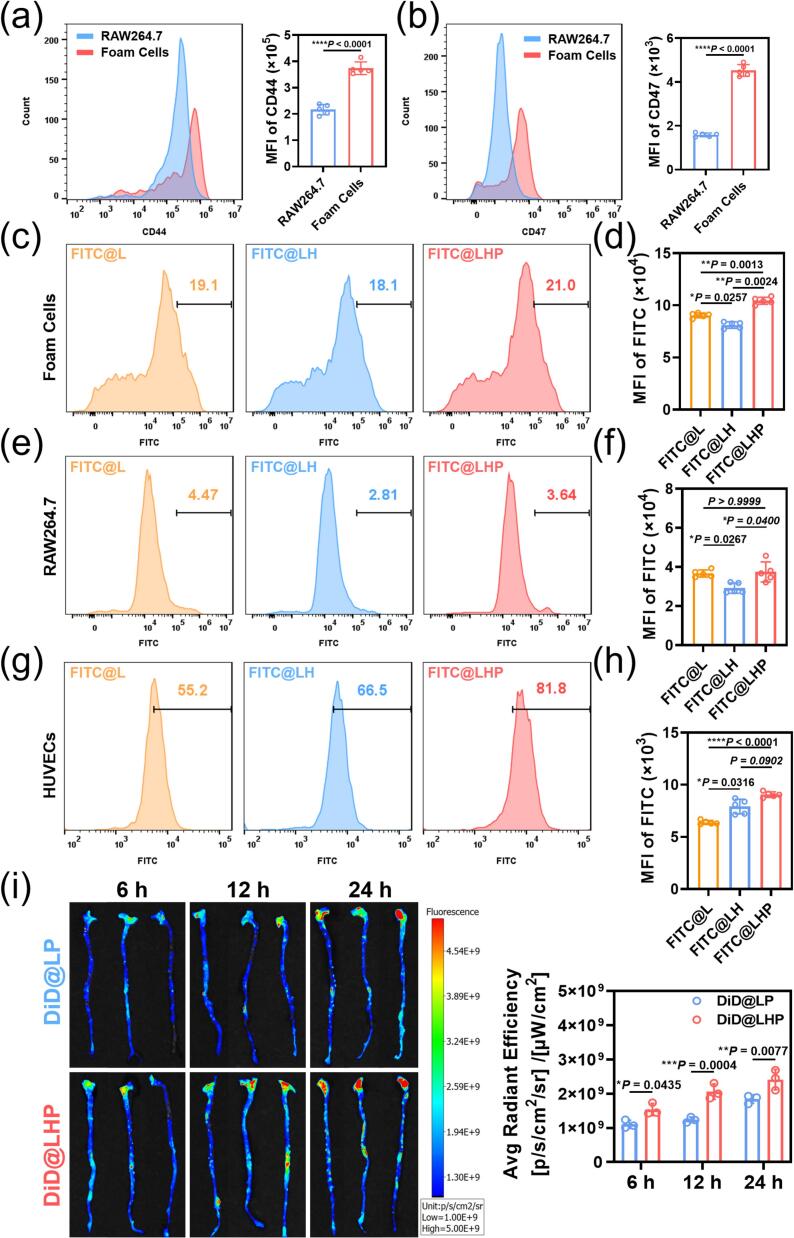
Targeting properties of atherosclerotic plaques *in vitro* and *in vivo*. (a, b) Flow cytometry analysis of CD44 (a) and CD47 (b) expression in RAW 264.7 and foam cells (induced by LPS and ox-LDL). (*n* = 5). (c-h) Flow cytometry analysis of the cellular internalization of different nanoparticles by foam cells (c), RAW 264.7 cells (e), and HUVECs (induced by LPS) (g) for 4 h and the mean fluorescence intensity (MFI) statistics in these cells (d), (f), and (h). (n = 5). (i) *Ex vivo* fluorescence imaging and quantitative analysis of the fluorescence intensity in the aorta in ApoE^−/−^ mice at 6, 12, and 24 h after intravenous administration of DiD@LP or DiD@LHP. (*n* = 3). The data are expressed as the means ± SDs. *P*-values: *P < 0.05, **P < 0.01, ***P < 0.001, and ****P < 0.0001.

The targets of lipid clearance and CD47 inhibition are foam cells, and thus we verified the targeting ability of Ato@LHP toward foam cells. CD44 and CD47 are highly expressed in foam cells (Fig. 2(a) and 2(b)) and facilitate the specific targeted delivery of Ato@LHP. We replaced atorvastatin with fluorescein isothiocyanate (FITC) to obtain FITC@L, FITC@LH, and FITC@LHP, which were used for subsequent cellular uptake experiments to study the targeting properties of the drug. Flow cytometry revealed that the degree of drug internalization was significantly greater in the FITC@LHP group than in the FITC@L group in foam cells after the induction of polarization *in vitro* (Fig. 2(c), 2(d), and Fig. S5). However, in the normal macrophage group without polarization induction, the degree of drug internalization in the FITC@LHP group was not significantly (*P* > 0.9999) different from that in the FITC@L group (Fig. 2(e), 2(f) and Fig. S5). These findings confirmed that FITC@LHP can be internalized to a greater extent by foam cells. Interestingly, we found that endocytosis efficiency was reduced in the FITC@LH group. This may be because the specific binding between HA ligands and CD44 receptors slows internalization (Rios de la Rosa et al., 2017; Rios de la Rosa et al., 2019). These findings indicate that CD44 expression can increase macrophage capture of HA-modified drugs but slow their internalization.

In addition, targeting damaged endothelial cells, which highly express CD44, is also important for atherosclerosis treatment, and thus LPS-stimulated human umbilical vein endothelial cells (HUVECs) were used to simulate damaged vascular endothelial cells at the plaque site to verify the targeting effect. Compared with those in the FITC@L group, cell uptake in the FITC@LH and FITC@LHP groups was significantly greater, and that in the FITC@LHP group was greater, which was due to the multiple types of endocytosis mediated by HA and Pep-20 in damaged vascular endothelial cells that highly expressed CD44 and CD47 (Fig. 2(g), 2(h) and Fig. S6) (Ma et al., 2023; Singh et al., 2023). Taken together, the above results demonstrate the excellent uptake of Ato@LHP by foam cells and damaged vascular endothelial cells, which is conducive to the realization of targeted drug delivery to plaques.

Next, we used ApoE^−/−^ mice fed a high-fat diet for 8 weeks as an *in vivo* model of atherosclerosis to evaluate the targeting ability of LHP. We prepared DiD@LP and DiD@LHP for *ex vivo* fluorescence imaging experiments. The results revealed that the fluorescence intensity of DiD@LHP in arterial tissues increased gradually over time and was greater than that of DiD@LP (Fig. 2(i)), but no significant difference was observed in the fluorescence intensity of the organs examined (Fig. S7). The aortic enrichment ratio was consistently higher than that of the major organs across all time points, peaking at 1.67 at 12 h (Fig. S8). In conclusion, the targeting effect of LHP is more conducive to the accumulation of drugs in plaque sites, which can improve the efficiency of drug delivery.

### 3.3 Therapeutic efficacy of Ato@LHP on foam cells *in vitro*

**Fig. 3:**
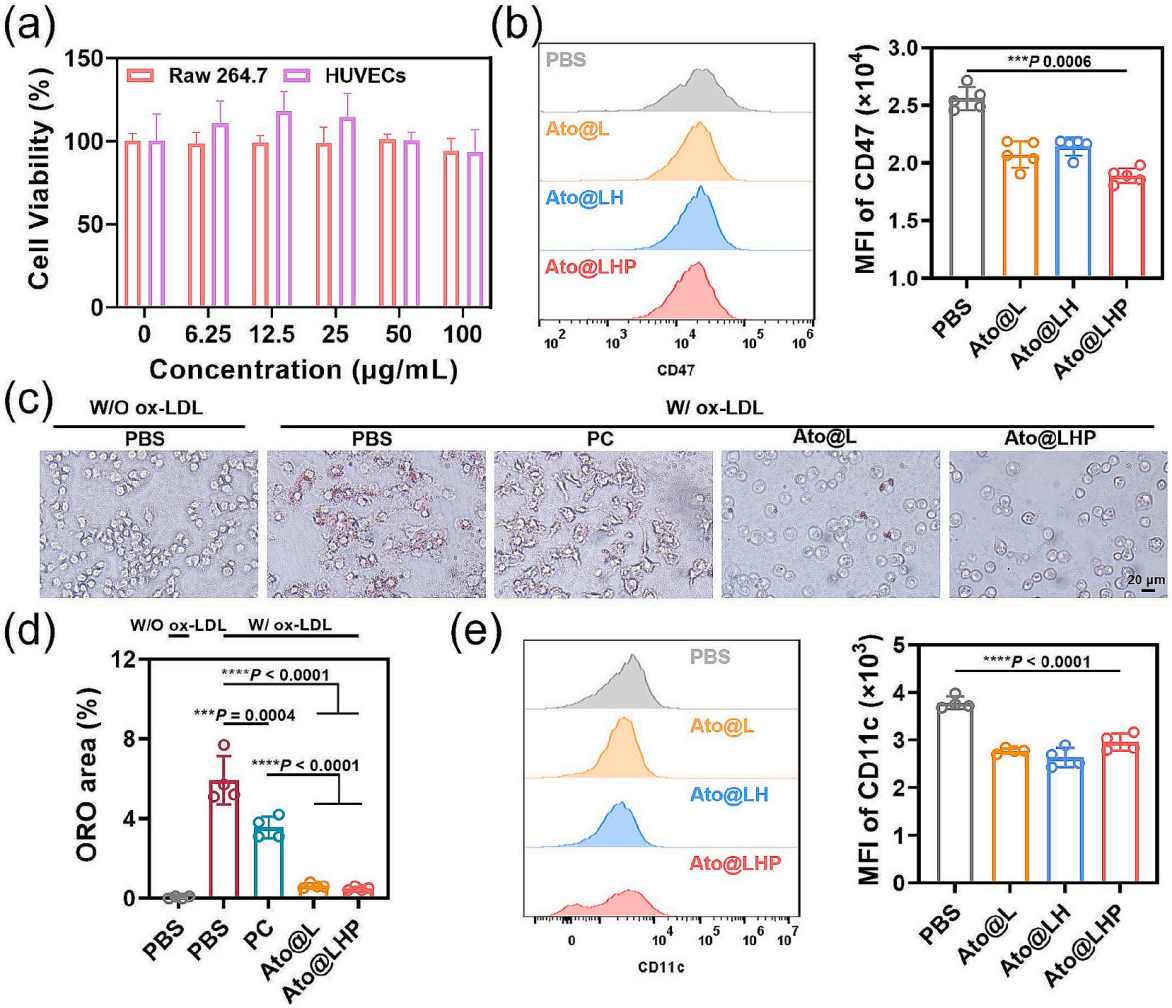
Therapeutic efficacy of Ato@LHP on foam cells *in vitro*. (a) CCK-8 assay of the viability of RAW 264.7 cells and HUVECs after treatment with different doses of Ato@LHP for 24 h. (*n* = 4). (b) Flow cytometry analysis and mean fluorescence intensity (MFI) statistics of CD47 expression in foam cells after different treatments for 24 h. (n = 5). (c) Images of lipid droplets (oil red O staining) in foam cells after different treatments for 24 h. Scale bar = 20 μm. (d) ORO area statistics of (d). (n = 4). (e) Flow cytometry analysis and mean fluorescence intensity (MFI) statistics of M1 polarization in RAW 264.7 cells after different treatments for 24 h. (n = 4). The data are expressed as the means ± SDs. *P*-values: *P < 0.05, **P < 0.01, ***P < 0.001, and ****P < 0.0001. (For interpretation of the references to colour in this figure legend, the reader is referred to the web version of this article.)

Before validating the therapeutic effect of Ato@LHP *in vitro*, we first assessed its cellular safety. The cytotoxicity of Ato@LHP on RAW 264.7 cells and HUVECs at different concentrations was observed *via* an *in vitro* cytotoxicity assay (CCK-8). The results revealed that the survival rates of RAW 264.7 cells and HUVECs were greater than 90% after 24 h of treatment with Ato@LHP (100 μg mL^−1^ atorvastatin), which shows that Ato@LHP had good biocompatibility (Fig. 3(a)). To verify the therapeutic effect of Ato@LHP, the regulatory effect of Ato@LHP on foam cells was evaluated *in vitro*. The results revealed the most significant decrease in CD47 expression in foam cells in the Ato@LHP group compared with the PBS group (Fig. 3(b)). Meanwhile, Fig. S9 showed that the LP group exerted a moderate downregulatory effect on CD47 expression, with a 16.6% reduction relative to PBS. Collectively, these findings demonstrate the synergistic effect of dual CD47 blockade mediated by Pep-20 peptide and atorvastatin. The downregulation of CD47 is beneficial for blocking the CD47-SIRPα pathway, which can restore the function of macrophages and facilitate the clearance of apoptotic foam cells more efficiently.

In addition, we investigated the ability of macrophages to inhibit the uptake of ox-LDL after different treatments and found that compared with those treated with PBS, macrophages treated with ox-LDL had minimal lipoprotein accumulation in the Ato@L and Ato@LHP groups. Interestingly, lipoprotein uptake was also decreased in the PC group (Fig. 3(c) and 3(d)). On one hand, phospholipid bilayers sequester cholesterol to solubilize extracellular free cholesterol crystals (Fig. S10). On the other hand, phospholipids internalized by cells remodel lipid trafficking pathways and inhibit ox-LDL uptake, thereby reducing intracellular lipid deposition (Gong et al., 2022). These findings suggest that phospholipids in Ato@LHP act synergistically with atorvastatin to inhibit lipoprotein uptake, which is beneficial for inhibiting the excessive accumulation of lipoproteins in macrophages and reducing the formation of foam cells in atherosclerotic plaques. In addition, we also found that atorvastatin treatment (Ato@L, Ato@LH, and Ato@LHP) inhibited the M1 polarization of macrophages (Fig. 3(e)), which helped alleviate inflammation at the plaque site (Caselli et al., 2022). In summary, Ato@LHP can effectively downregulate CD47 in foam cells, which further promotes the restoration of macrophage efferocytosis, inhibits the accumulation of lipids in macrophages and reduces M1-type polarization.

### 3.4 Transcriptomic mechanism underlying the regulation of foam cells by Ato@LHP

To elucidate the transcriptional regulatory mechanisms underlying the therapeutic effect of Ato@LHP against atherosclerosis, we performed bulk RNA-seq on foam cells (RAW264.7) treated with PBS or Ato@LHP to profile transcriptomic changes. Principal component analysis (PCA) revealed a clear separation between the two groups (Fig. S11(a)), confirming that Ato@LHP induced substantial transcriptomic alterations in foam cells.

Subsequently, functional enrichment analyses were conducted. KEGG pathway analysis showed that differentially expressed genes (DEGs) were primarily enriched in inflammation and atherosclerosis-related signaling pathways (such as the TNF, chemokine, and PI3K-Akt pathways), as well as pathways involved in apoptosis, efferocytosis, and actin cytoskeleton regulation. Meanwhile, Gene Ontology (GO) analysis demonstrated that these DEGs were markedly enriched in immune and inflammatory processes, including leukocyte migration, chemotaxis, and leukocyte activation (Fig. S11(b) and S11(c)).

To validate these findings, we examined the expression of key representative genes. In terms of inflammatory pathways, Ato@LHP treatment significantly downregulated pro-inflammatory genes including *IL1b*, *IL6*, *Ccl2*, *Nos2*, and *Cxcl10* (Fig. S11(d)), while upregulating anti-inflammatory and atheroprotective genes including *Tgfb1*, *Klf2*, *Klf4*, *Tnfaip3*, and *Mrc1* (Fig. S11(e)). Notably, regarding efferocytosis-related genes (Fig. S11(f)), Ato@LHP significantly decreased the expression of *CD47* and *SIRPa*, while increasing the levels of *Mertk*, *Mfge8*, and *Itgb3*. This suggests that Ato@LHP inhibits the endogenous transcription of *CD47* in foam cells, thereby blocking the “don't eat me” signal and restoring efficient efferocytosis. Furthermore, in terms of the apoptosis pathway, pro-apoptotic genes including *Fas*, *Bax*, and *Bid* were downregulated, whereas anti-apoptotic genes including *Mcl1* and *Xiap* were upregulated (Fig. S11(g)). Collectively, these results demonstrate that Ato@LHP promotes an anti-inflammatory, pro-efferocytotic, and anti-apoptotic phenotype in foam cells, which likely contributes to its anti-atherosclerotic effects.

### 3.5 Therapeutic efficacy of Ato@LHP on atherosclerosis *in vivo*

**Fig. 4:**
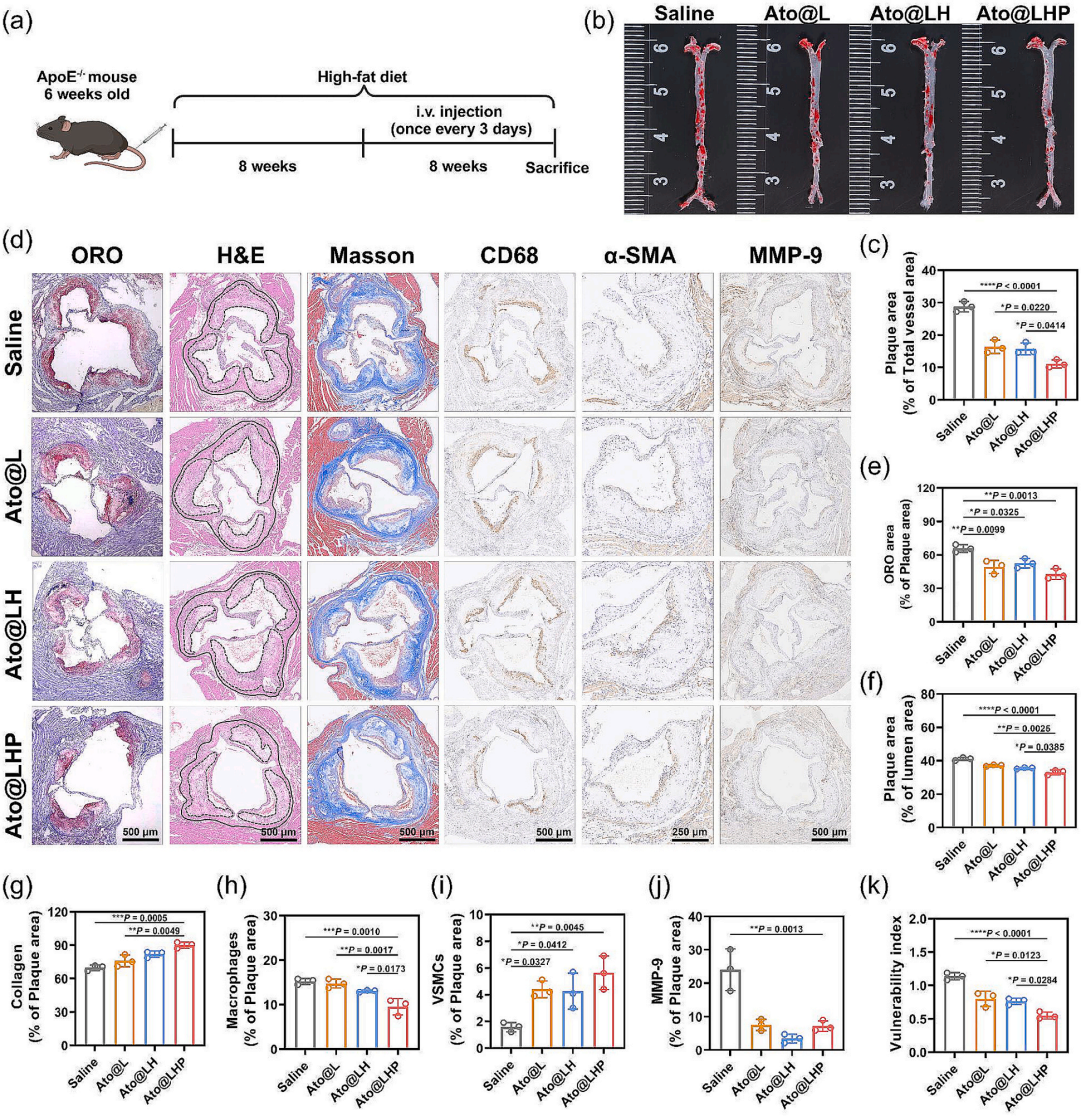
Therapeutic efficacy of Ato@LHP in atherosclerosis *in vivo*. (a) Schematic of the atherosclerosis model construction and treatment protocol for ApoE^−/−^ mice. (b) Oil red O staining and (c) quantitative statistics of whole aortas from atherosclerotic mice after different treatments. (n = 3). (d) Aorta root sections were stained with ORO, H&E, and Masson's trichrome as well as with anti-CD68, anti-α-SMA and anti-MMP-9 antibodies after different treatments. Quantitative analysis of the (e) ORO area of plaques, (f) plaque area within the lumen, (g) collagen within plaques, (h) macrophages in plaques, (i) VSMCs in plaques, and (j) MMP-9 in plaques. (k) Analysis of the vulnerability index of plaques. (n = 3). The data are expressed as the means ± SDs. *P*-values: *P < 0.05, **P < 0.01, ***P < 0.001, and ****P < 0.0001. (For interpretation of the references to colour in this figure legend, the reader is referred to the web version of this article.)

We further verified the therapeutic effect of Ato@LHP in atherosclerotic mice. Six-week-old male ApoE^−/−^ mice were randomly divided into the saline, Ato@L, Ato@LH, and Ato@LHP groups after 8 weeks of high-fat feeding and subjected to tail vein injection every 3 days for 8 weeks, during which the high-fat diet was maintained (Fig. 4(a)). To assess lipid deposition in the aorta, oil red O staining was performed on the entire aorta, where the red area represents the plaque lesion (Fig. 4(b)). Compared with saline, Ato@LHP treatment reduced the plaque area from 28.8% to 11.1% (Fig. 4(c)), and less lipid accumulation was also observed *via* oil red O staining of the arterial roots (42.7% ± 3.8%, *P* = 0.0013) (Fig. 4(d) and 4(e)). To further observe atherosclerotic plaque development, plaque area (haematoxylin and eosin (H&E) staining), vulnerability Masson's trichrome (Masson) staining and staining for CD68, alpha smooth muscle actin (α-SMA), and matrix metalloproteinase-9 (MMP-9) were performed (Fig. 4(d)). H&E staining revealed that, compared with the saline group, Ato@LHP significantly reduced the proportion of plaque area in the lumen from 41.1% ± 0.7% to 33.1% ± 1.2% (Fig. 4(f)). More collagen (90.1% ± 2.2%, *P* = 0.0005) was observed by Masson's trichrome staining (Fig. 4(g)) in the Ato@LHP group than in the other groups, which indicates that plaques were more stable in this group. CD68 staining revealed that the percentage of macrophages decreased from 15.2% ± 0.5% in the saline group to 9.5% ± 1.5% in the Ato@LHP group (Fig. 4(h)). The results of α-SMA staining revealed that, compared with the saline group (1.6% ± 0.3%), the number of smooth muscle cells in the Ato@LHP (5.6% ± 1.0%) group was significantly greater, which indicates that Ato@LHP treatment can promote smooth muscle cell proliferation (Fig. 4(i)). Matrix metalloproteinase 9 (MMP-9) is mainly secreted by foam cells in plaques and can degrade the extracellular matrix at plaque sites, leading to plaque instability (Kadikoylu et al., 2008). MMP-9 staining revealed that Ato@LHP significantly reduced foam cell production and MMP-9 secretion from 24.0% ± 5.1% to 7.1% ± 1.3% (Fig. 4(j)). Taken together, these results indicate that the stability of plaques was increased after Ato@LHP treatment and that the vulnerability index of plaques decreased from 1.14 ± 0.05 to 0.55 ± 0.04 (Fig. 4(k)). Therefore, Ato@LHP can effectively remove lipids from plaques and increase their stability, which is conducive to the alleviation of atherosclerosis.

### 3.6 Therapeutic mechanism of Ato@LHP in atherosclerosis

**Fig. 5:**
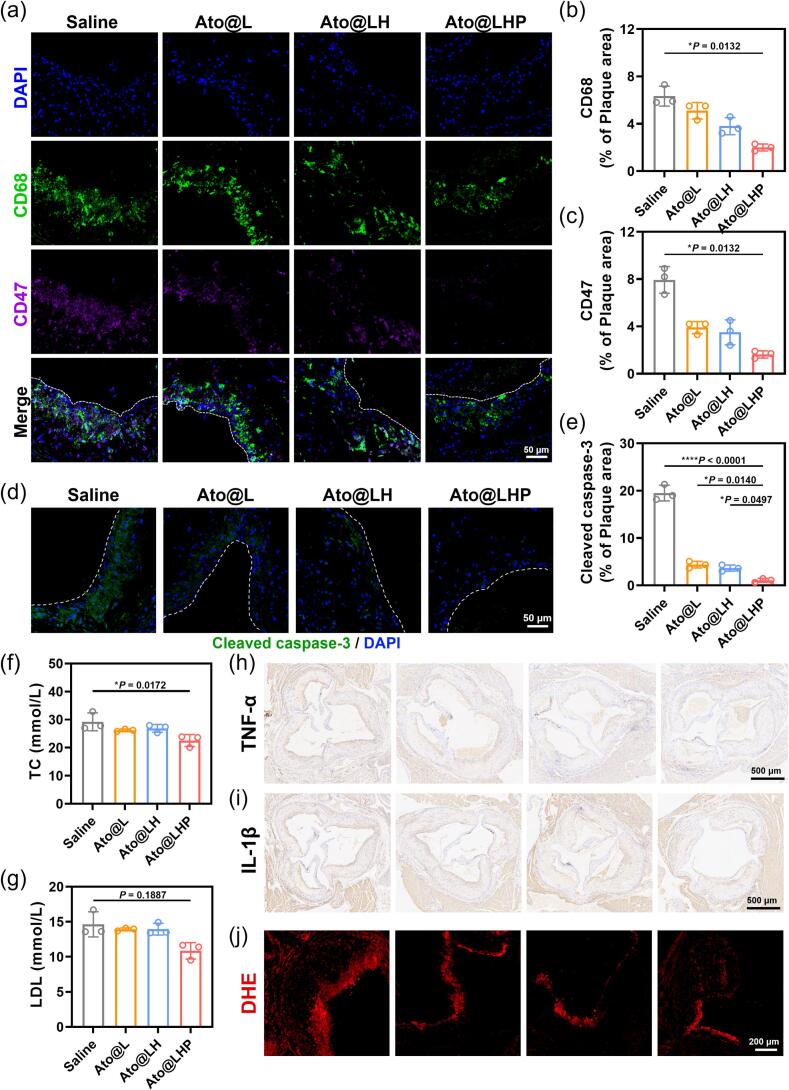
Therapeutic mechanism of Ato@LHP in atherosclerosis. (a) Immunofluorescence staining and quantitative analysis of (b) CD68 and (c) CD47 in aortic roots after the different treatments. (n = 3). (d) Immunofluorescence staining and (e) quantitative analysis of cleaved caspase-3 in aortic roots after the different treatments. (n = 3). Serum levels of (f) TC and (g) LDL after the different treatments. (n = 3). (h) TNF-α and (i) IL-1β staining of the aortic root after the different treatments. (n = 3). (j) Immunofluorescence staining of aortic roots by DHE after the different treatments. (n = 3). The data are expressed as the means ± SDs. *P*-values: *P < 0.05, **P < 0.01, ***P < 0.001, and ****P < 0.0001.

To further investigate the therapeutic mechanism of Ato@LHP in atherosclerosis *in vivo*, we performed immunofluorescence staining for CD68 and CD47 in aortic root sections. Consistent with the immunohistochemical results, Ato@LHP treatment significantly reduced the number of macrophages in plaques (Fig. 5(a) and 5(b)). Furthermore, Ato@LHP treatment significantly reduced the expression of CD47 at the plaque site (Fig. 5(a)), and specifically, Ato@LHP was more effective than Ato@L (Fig. 5(c)) because of the dual-blockade effect on CD47. Moreover, more apoptotic cells were cleared from plaques in the Ato@LHP group (Fig. 5(d) and 5(e)), which was attributed to the promotion of efferocytosis due to CD47 downregulation.

In addition, we evaluated blood lipids and found that the serum cholesterol and low-density lipoprotein levels in the Ato@LHP group were reduced (Fig. 5(f) and 5(g)), which means that Ato@LHP can reduce the accumulation of low-density lipoprotein at plaque sites, thereby inhibiting foam cell formation and the development of atherosclerosis.

Moreover, we observed that proinflammatory cytokines (TNF-α and IL-1β) were downregulated in the Ato@LHP group compared with the saline group (*P* = 0.0021 and *P* = 0.0242 for TNF-α and IL-1β, respectively) (Fig. 5(h), 5(i) and Fig. S12). This can be attributed to the anti-inflammatory effect of atorvastatin, along with the resolution of inflammation resulting from enhanced efferocytosis. Moreover, we also observed less ROS production in the Ato@LHP group compared with the saline group (Fig. 5(j) and Fig. S13), indicating an improvement in the inflammatory microenvironment. Therefore, Ato@LHP can restore the efferocytosis of macrophages *via* dual blockade of CD47, which would further promote the clearance of apoptotic cells and enhance lipid clearance and anti-inflammatory ability to achieve a therapeutic effect in atherosclerosis.

### 3.7 Biosafety assessment of Ato@LHP

Biosafety is a prerequisite for clinical application; therefore, we systematically evaluated the biosafety of Ato@LHP *in vivo*. After 8 weeks of treatment, no significant difference was observed in the weights of the mice in each group (Fig. S14). In addition, atorvastatin treatment may cause liver function impairment and blood glucose disorders (Andrade et al., 2006; Björnsson et al., 2012; Kim et al., 2024); therefore, we investigated liver function and blood glucose indices, and compared with the saline group, the other groups presented no significant differences (Fig. S15). Moreover, H&E staining of tissue from major organs also revealed no obvious tissue necrosis or pathological abnormalities after treatment with different drugs (Fig. S16). To evaluate hematological safety of CD47 blockade, complete blood count (CBC) tests were performed on 6-week-old male C57BL/6 J mice receiving Ato@LHP at a 2.5-fold higher dose (every other day) to match the cumulative exposure of the therapeutic regimen. As shown in Fig. S17, all hematological parameters related to erythrocytes and platelets revealed no statistically significant differences between the Ato@LHP and Saline groups, and all values remained within the normal physiological reference range of mice. The above experimental data collectively indicate that Ato@LHP has a good biosafety profile for the treatment of atherosclerosis.

## 4 Discussion

Our findings demonstrate that Ato@LHP achieves dual-targeted delivery of atorvastatin and Pep-20 to plaque foam cells through passive and LHP-mediated active targeting. At the lesion site, ROS triggers TK linker cleavage to release Pep-20 for exogenous CD47 blockade, while internalized atorvastatin suppresses CD47 transcription endogenously. This dual blockade restores efferocytosis of apoptotic foam cells, an effect further reinforced by atorvastatin- and phospholipid-mediated lipid clearance and anti-inflammatory actions, collectively reducing plaque burden in the early-stage mouse model.

Nevertheless, several limitations of the present study should be noted. First, while *ex vivo* fluorescence imaging provided semi-quantitative biodistribution data, future studies incorporating LC-MS/MS quantification will be essential to precisely determine drug exposure at the target site. Second, our transcriptomic analysis was performed on *in vitro*-induced foam cells; extending this to plaque macrophages *in vivo* would better capture the transcriptional landscape within the actual atherosclerotic microenvironment. Third, the absence of a non-responsive (TK-deleted) carrier control and a free atorvastatin plus free Pep-20 combination group in the current design means that further refinement of control groups is needed to fully dissect the contributions of responsive release and nanocarrier synergy. Fourth, given that our animal experiments were confined to male mice with early-stage plaques (8-week HFD), expanding the evaluation to female mice and more advanced, unstable lesion models will be important to assess the translational potential of this strategy. These considerations point toward future work involving pharmacokinetic quantification, *in vivo* tissue-level molecular profiling, optimized control designs, and clinically relevant lesion models to further substantiate and extend our current findings.

## 5 Conclusion

In conclusion, we have successfully constructed a ROS-responsive nanoplatform, Ato@LHP, which promotes the elimination of apoptotic foam cells in an early-stage atherosclerotic mouse model through a dual mechanism combining CD47 blockade and lipid clearance. This study demonstrates that a synergistic therapeutic strategy based on active targeting and responsive release can significantly reduce plaque burden, offering a new approach for atherosclerosis therapy.

## CRediT authorship contribution statement

**Peng Yuan:** Writing – original draft, Visualization, Conceptualization. **Shibo Tian:** Visualization, Data curation, Conceptualization. **Chao Cui:** Investigation, Formal analysis, Data curation. **Chenglu Sun:** Investigation, Formal analysis, Data curation. **Chuang Tang:** Investigation, Formal analysis. **Dengfeng Gao:** Investigation, Formal analysis. **Lu Lu:** Resources, Investigation. **Keren Long:** Resources, Investigation. **Funeng Xu:** Resources, Conceptualization. **Gang Shu:** Resources, Data curation. **Wei Zhang:** Resources, Data curation. **Xiaoxia Liang:** Resources, Data curation. **Fanli Kong:** Resources, Funding acquisition. **Mingzhou Li:** Writing – review & editing, Supervision, Funding acquisition. **Haohuan Li:** Writing – review & editing, Supervision, Funding acquisition.

## Ethics statement

The animal experiments were conducted in accordance with the Laboratory Animal Management Committee of Sichuan Province (laboratory animal use licence number: SYXK (Chuan) 2020–224). All animal procedures were approved by the institutional animal ethics committee with the specific experimental protocol approval number of 2022303098, and the approval date was May 9, 2024. All animal operations strictly followed the ARRIVE guidelines.

## Funding

This work was supported by the National Key R & D Program of China (2023YFD1300400 to F.K.), the 10.13039/501100001809National Natural Science Foundation of China (32372846 and 32341051 to H.L., 32421005, 32494802 and 32225046 to M.L.), the Science and Technology Projects of Xizang Autonomous Region of China (XZ202501ZY0147 to M.L.).

## Declaration of competing interest

The authors declare that they have no known competing financial interests or personal relationships that could have appeared to influence the work reported in this paper.

All the contributing authors no conflict of interests in this work.

## Data Availability

The data that support the findings of this study are available from the corresponding author upon reasonable request. The bulk RNA-seq data in this study have been deposited in the NCBI Sequence Read Archive (SRA) under the BioProject accession number PRJNA1431201.
