# Supplementary material for: A ROS‑responsive liposome for atherosclerosis treatment via combined regulation of lipid metabolism and CD47 blockade
Source: Int J Pharm X. 2026 Jul 30;12:100626. doi: 10.1016/j.ijpx.2026.100626 (PMC13463256; doi:10.1016/j.ijpx.2026.100626)
Supplement: Supplementary file 1 [file mmc1.docx]

**Supplementary data**

**A ROS-responsive liposome regulates lipid metabolism combined with CD47 blocking for atherosclerosis treatment.**

Peng Yuan^1^, Shibo Tian^1^, Chao Cui^1^, Chenglu Sun^1^, Chuang Tang^2^, Dengfeng Gao^2^, Lu Lu^2^, Keren Long^2^, Funeng Xu^3^, Gang Shu^3^, Wei Zhang^3^, Xiaoxia Liang^3^, Fanli Kong^4^, Mingzhou Li^2^✉, and Haohuan Li^1^✉

*^1^ State Key Laboratory of Swine and Poultry Breeding Industry, College of Veterinary Medicine, Sichuan Agricultural University, Chengdu 611130, China*

*^2^ Livestock and Poultry Multi-omics Key Laboratory of Ministry of Agriculture and Rural Affairs, College of Animal Science and Technology, Sichuan Agricultural University, Chengdu 611130, China*

*^3^ College of Veterinary Medicine, Sichuan Agricultural University, Chengdu 611130, China*

*^4^ State Key Laboratory of Swine and Poultry Breeding Industry, College of Life Science, Sichuan Agricultural University, Chengdu 611130, China*

✉Address correspondence to Haohuan Li. lihaohuan@sicau.edu.cn; Mingzhou Li. mingzhou.li@sicau.edu.cn

Supplementary Material and Methods

**Materials**

Soya bean lecithin (PC) was purchased from Macklin Biochemical Technology Co., Ltd. (Shanghai, China). Atorvastatin was purchased from Xinweichuang Biotechnology (Chongqing) Co., Ltd. (Chongqing, China). DSPE-PEG-HA (MW: PEG2000, HA35000) and DSPE-PEG-TK-MAL (MW: PEG2000) were purchased from Ruixi Biotechnology Co., Ltd. (Xian, China). Pep-20 (CAWSATWSNYWRH) was purchased from Scier Biotechnology Co., Ltd. (Hefei, China). FITC and DiD were purchased from Beyotime Biotechnology Co., Ltd. (Shanghai, China). LPS and Oil Red O were purchased from Aladdin Biotechnology Co., Ltd. (Shanghai, China). Ox-LDL was purchased from Yiyuan Biotechnology Co., Ltd. (Guangzhou, China). Alexa Fluor® 488-conjugated anti-mouse/human CD44, Alexa Fluor® 647-conjugated anti-mouse CD47, and PE-conjugated anti-mouse CD11c antibodies were purchased from Biolegend (USA). The CD16/CD32 monoclonal antibody (93) manufactured by eBioscience™ was purchased from Thermo Fisher Scientific–CN Co., Ltd. (China). The anti-alpha smooth muscle actin (α-SMA) antibody was purchased from Abcam (USA). Anti-CD68 and anti-cleaved caspase-3 antibodies were purchased from Service Biotechnology Co., Ltd. (Wuhan, China). The MMP-9 antibody was purchased from Proteintech Group Biological Technology Co., Ltd. (Wuhan, China). The anti-TNF alpha/TNFA antibody was purchased from BOSTER Biological Technology Co., Ltd. (Wuhan, China). Anti-IL-1β was purchased from Bioss Biological Technology Co., Ltd. (Beijing, China). The Cell Counting Kit-8 (CCK-8) and dihydroethidium (DHE) were purchased from Biosharp Biotechnology Co., Ltd. (Hefei, China). Dulbecco's Modified Eagle’s Medium (DMEM) was purchased from Thermo Fisher Scientific – CN Co., Ltd. (China). Foetal bovine serum (FBS) was purchased from New Zealand.

**Cell Culture**

RAW 264.7 cells and HUVECs were obtained from the Shanghai Cell Bank, Chinese Academy of Sciences. The cells were cultured in 90% DMEM supplemented with 10% FBS and 1% penicillin/streptomycin at 37 °C in 5% CO_2_.

**Animals**

The ApoE^-/-^ mice (6 weeks old) were purchased from Shulaibao (Wuhan) Biotechnology Co., Ltd. (Wuhan, China). High-fat chow was purchased from Xietong Pharmaceutical Bioengineering Co., Ltd. (Jiangsu, China).

***In Vitro* Drug Release**

Drug release capacity was assessed using the dialysis method. Briefly, 2 mL of liposomes was added to a dialysis bag (MW = 2000), which was then immersed in 30 mL of release medium (1×PBS, pH 7.4, with 0.1% (W/O) Tween 80) at 37 °C with stirring at 100 rpm. At different time points, 1 mL of the release sample was removed, and 1 mL of the same release medium was added. Finally, the drug content of the released samples at each time point was detected *via* a UV‒Vis spectrophotometer, and the cumulative release rate was calculated.

***In Vitro* Stability Testing**

Liposome stability was assessed by measuring the particle size and PDI of the liposomes stored at 4 °C for 10 days, during which the changes were measured every 2 days.

**ROS-Triggered Peptide Release**

After coincubation of Ato@LHP with 1× PBS (pH 7.4) or 1× PBS containing hydrogen peroxide (pH 7.4, with 1 mM H_2_O_2_) at 37 °C, changes in the Pep-20 concentration were observed by HPLC, and morphological changes were observed by transmission electron microscopy (TEM).

***In Vitro* Cholesterol Solubilization**

Excess cholesterol was added to phosphate-buffered saline (PBS) and PBS containing PC at 37 °C for 24 h. The undissolved cholesterol was filtered off with a 0.45 μm filter to obtain the cholesterol solution. The cholesterol content was measured by HPLC at a detection wavelength of 205 nm.

**Cell Cytotoxicity Evaluation**

RAW 264.7 cells and HUVECs were seeded in 96-well plates (1×10^4^ cells per well). After 24 hours of culture, the cells were treated with media containing different doses of Ato@LHP, and the cell viability was measured *via* the CCK-8 method after 24 hours of incubation.

***In vitro* Oil Red O Staining Assay**

RAW 264.7 cells were seeded in 6-well plates. The RAW 264.7 cell group was not treated, while the foam cell group was incubated with 40 μg mL^-1^ ox-LDL 200, ng mL^-1^ LPS and different drugs (PC, Ato@L, or Ato@LHP) for 24 h. Oil red O staining was subsequently performed with a newly formulated ORO working solution.

**Bulk RNA-seq analysis**

RAW 264.7 cells were seeded in 6-well plates and induced with 40 μg mL^-1^ ox-LDL and 200 ng mL^-1^ LPS, and different groups were treated with PBS or Ato@LHP for 24 h. Library preparation and high-throughput RNA sequencing were performed by LC-Bio Technology Co., Ltd. (Hangzhou, zhejiang, China)

Supplementary Figures.

|  | **EE (%)** | **LE (%)** |
| --- | --- | --- |
| **Ato@L** | 75.81 ± 1.52 | 6.89 ± 0.14 |
| **Ato@LH** | 72.64 ± 0.69 | 5.93 ± 0.06 |
| **Ato@LHP** | 82.95 ± 0.84 | 5.18 ± 0.05 |

**Table S1** EE% and LE% data of Ato@L, Ato@LH, and Ato@LHP. Data are expressed as the mean ± SD.

**Figure S1** HPLC results of 1 mM H_2_O_2_, Free Pep-20, Ato@LHP, and Ato@LHP + 1 mM H_2_O_2_ at a wavelength of 214 nm.

**Figure S2** Standard curve of the Pep-20 and graft ratio in Ato@LHP. Data are expressed as the mean ± SD.

**Figure S3** DLS particle size and PDI data (a) and (b) for Ato@L and Ato@LH. Data are expressed as the mean ± SD.

**Figure S4** TEM images (a) and (b) of Ato@L and Ato@LH, scale bar = 100 nm.

**Figure S5** Quantitative analysis by flow cytometry of FITC^+^ cells in RAW 264.7 and foam cells after treatment with different nanoparticles. (n = 5). Data are expressed as the mean ± SD. *P*-values: **P* < 0.05, ***P* < 0.01, ****P* < 0.001, and *****P* < 0.0001.

**Figure S6** Quantitative analysis by flow cytometry of FITC^+^ cells in HUVECs (induced by LPS) after treatment with different nanoparticles. (n = 5). Data are expressed as the mean ± SD. *P*-values: **P* < 0.05, ***P* < 0.01, ****P* < 0.001, and *****P* < 0.0001.

**Figure S7** (a) Ex vivo fluorescence imaging and (b) quantitative analysis of fluorescence intensity of the heart, liver, spleen, lung, and kidney in ApoE^-/-^ mice at 6, 12, and 24 h after intravenous administration of DiD@LP and DiD@LHP. (n = 3). Data are expressed as the mean ± SD. *P*-values: **P* < 0.05, ***P* < 0.01, ****P* < 0.001, and *****P* < 0.0001.

**Figure S8** Relative enrichment ratio of Ato@LHP compared with Ato@LP in aorta and total major organs (Heart, Liver, Spleen, Lung, and Kidney) at 6, 12, and 24 h post intravenous injection.

**Figure S9** Quantitative flow cytometry analysis of CD47 expression in foam cells after 24 h treatment with PBS, LP (Pep20-only liposomes), or Ato@LHP. (n = 4). Data are expressed as the mean ± SD. *P*-values: **P* < 0.05, ***P* < 0.01, ****P* < 0.001, and *****P* < 0.0001.

**Figure S10** Solubility of cholesterol crystals after 24 h of PC incubation. (n = 4). Data are expressed as the mean ± SD. *P*-values: **P* < 0.05, ***P* < 0.01, ****P* < 0.001, and *****P* < 0.0001.

**Figure S11** Bulk RNA-seq analysis in foam cells (RAW264.7) treated with PBS or Ato@LHP. (n = 4). (a) Principal component analysis (PCA) of global gene expression profiles. (b) KEGG pathway enrichment analysis of differentially expressed genes (DEGs). (c) GO enrichment analysis of DEGs. Relative mRNA expression of pro-inflammatory genes (d), anti-inflammatory genes (e), phagocytosis-related genes (f), and apoptosis-related genes (g). DEGs were identified with adjusted *P*-value < 0.05. Data are mean ± SD. **P* < 0.05, ***P* < 0.01, ****P* < 0.001, *****P* < 0.0001.

**Figure S12** Quantitative analysis of (a) TNF-α and (b) IL-1β staining. (n = 3). Data are expressed as the mean ± SD. *P*-values: **P* < 0.05, ***P* < 0.01, ****P* < 0.001, and *****P* < 0.0001.

**Figure S13** Quantitative analysis of DHE immunofluorescence staining. (n = 3). Data are expressed as the mean ± SD. *P*-values: **P* < 0.05, ***P* < 0.01, ****P* < 0.001, and *****P* < 0.0001.

**Figure S14** Body weight changes in mice during different treatments. (n = 10).

**Figure S15** Serum levels of (a) AST, (b) ALT and (c) Glu after the different treatments. (n = 3). The data are expressed as the means ± SDs. *P*-values: **P* < 0.05, ***P* < 0.01, ****P* < 0.001, and *****P* < 0.0001.

**Figure S16** H&E staining of major organs (heart, liver, spleen, lung, and kidney) after the different treatments. (n = 3).

**Figure S17** Hematological safety evaluation of Ato@LHP in healthy mice after treatment. (n = 5). (a) Red blood cell count (RBC); (b) Hemoglobin concentration (HGB); (c) Hematocrit (HCT); (d) Mean corpuscular volume (MCV); (e) Platelet count (PLT); (f) Platelet distribution width (PDW); (g) Plateletcrit (PCT); (h) Mean platelet volume (MPV). Data are presented as mean ± SD.
